# MicroRNA Era: The Importance for Diagnosis and Prognosis of Adrenocortical Tumors

**DOI:** 10.1155/2014/381917

**Published:** 2014-06-23

**Authors:** João Evangelista Bezerra, Ana Claudia Latronico

**Affiliations:** ^1^Instituto do Câncer do Estado de São Paulo (ICESP), 05403-000 Sao Paulo, SP, Brazil; ^2^Laboratório de Hormônios e Genética Molecular LIM/42 do Hospital das Clínicas, Disciplina de Endocrinologia e Metabologia da Faculdade de Medicina da Universidade de São Paulo, 05403-000 Sao Paulo, SP, Brazil

## Abstract

MicroRNAs play an essential role in posttranscriptional regulation of gene expression. They are evolutionary conserved, small, noncoding, 19–22-nucleotide RNAs, whose abnormalities, such as up- or downregulated expression, have been associated with several neoplasms, including adrenocortical tumors. Expression levels of distinct microRNAs can distinguish benign from malignant adrenal tumors. This current review provides recent data on the miRNAs profile in benign and malignant adrenocortical tumors diagnosed in adult and pediatric patients.

## 1. Introduction

Adrenocortical tumors are common entities [1]. Autopsy studies estimate a prevalence ranging from 1.4 to 2.9% and reaching up to 9% in elderly patients. Incidental adrenal tumors, when adrenal lesions are found during image tests for other reasons, have been found with increasing frequency [1, 2]. The primary adrenocortical carcinoma (ACC), however, is a rare entity. It has an estimated annual incidence of 0.5 to 2 cases per million people and accounts for 0.02% of all cancers [3, 4]. ACC has a bimodal peak of incidence, the first in children less than 5 years old and the second in adults in their fourth or fifth decade of life. It is more common in women than in men with a ratio of 1.5 : 1 [5]. ACC often causes endocrine disorders. Approximately, 60% of ACCs in adults present as a well-defined hormonal syndrome. Cushing syndrome (45%), mixed Cushing and virilization (25%), and isolated virilization syndrome (10%) are the most frequent presentations [6–9]. In childhood, ACC usually presents as virilization syndrome (84%) and less frequently as isolated Cushing syndrome (6%) [10, 11]. Patients who have nonfunctioning ACC present with signs and symptoms related to tumor growth, such as abdominal pain [6, 8].

Because of the huge difference in prognosis and treatment, an accurate distinction between adrenocortical adenomas (ACA) and ACCs is very important. The differentiation between ACA and ACC is primarily based on macroscopic and microscopic features. Some histological score systems were developed to improve accuracy [12–14]. The Weiss score system is the most widely used [15]. It analyzes nine histological parameters associated with adrenocortical neoplasms that metastasized or recurred locally: three related to structure features of the tumor (confluent necrosis, diffuse architecture, and quantity of clear cell component), three related to cytological features (nuclear atypia, atypical mitosis, and number of mitoses), and three related to tumor invasiveness (vascular invasion, sinusoidal invasion, and invasion of adrenal capsule). This system classifies as ACC any tumor that exhibits at least three of the nine features. Unfortunately, even experienced pathologists sometimes are unable to clearly define an adrenocortical tumor as malignant or benign [16]. Also, an adrenocortical tumor Weiss score of 2 or 3 may have unpredictable behavior. There are reports of adrenocortical tumor with Weiss score 2 that behaves in a malignant manner while many tumors with Weiss score 3 are cured by surgery alone. In addition, the applicability of Weiss criteria in the pediatric population is restricted due to poor correlation between histological findings and clinical evolution in this population [15, 17–19].

Adrenocortical carcinoma is potentially fatal. The five-year overall survival ranges from 35 to 58% in the more recent series [7, 9, 20, 21]. Stage is the most important prognostic factor, with a more advanced stage correlating with less durable survival rate [22, 23]. Unfortunately, in almost 70% of patients, the disease has spread beyond the adrenal gland at the time of diagnosis [24, 25]. Complete surgical resection of adrenal limited disease is the only potentially curative treatment procedure. Therapeutic options for more advanced diseases remain restricted. Mitotane (o,p′-DDD) is an isoform of the insecticide dichlorodiphenyltrichloroethane (DDT) and is the only approved drug for ACC treatment. It is used for hormonal blockage of functional advanced diseases and is a direct antitumor activity drug [26–29]. However, mitotane is a difficult drug to manage because of its narrow therapeutic index and important side effects [28, 29]. Cytotoxic chemotherapy and/or radiation therapy have been used in the advanced setting but with limited responses [30]. Even the new classes of target therapies have not been useful for improving survival [31–34].

## 2. MicroRNA Function and Carcinogenesis

MicroRNAs (miRNA) are a class of noncoding RNAs of 19–22 nucleotides in length. They specifically bind the 3′-untranslated regions (3′UTR) of the messenger RNA (mRNA) molecule inducing their degradation or inhibiting their translation, causing partial or full silencing of the respective protein-coding gene [35]. The miRNAs are involved in the regulation of several physiological processes in almost all eukaryotes, including development, growth, differentiation, and metabolism. Therefore, the involvement of miRNAs as an important player in carcinogenesis was not surprising. The first evidence of miRNA involvement in neoplastic processes came from the observation that these genes are frequently located at fragile regions of the chromosomes [36]. However, it was only in 2002 that Calin et al. [37] reported the abnormal expression of miRNA in a human neoplasm. Patients diagnosed with chronic lymphocytic leukemia and deletion of* locus* 13q14 presented low expression of two miRNAs, miR-15 and miR-16. Furthermore, two patients also presented germline mutation of one* locus* of miR-16-1 gene and loss-of-heterozygosity in the chronic lymphocytic leukemia cells, fulfilling the Knudson model of inactivation of a tumor-suppressor gene [38]. Lu et al. [39] analyzed the miRNA expression profile from multiple tumor samples. They demonstrated that miRNA expression profile was able to put together different tumor samples sharing the same development linage and to classify poor differentiated tumors based on their origins with a better correlation than mRNA profile performed in the same samples. Since then, several studies have reported miRNA expression profiles abnormalities in most variety of tumors. The miRNA profiles were able to classify tumors based on prognosis, chance of recurrence, or response to therapy [40]. In addition, miRNAs are particularly resistant to degradation from the widely spread RNAase enzymes [41]. Because of that, miRNAs are easily isolated from formalin-fixed paraffin-embedded tissue samples [42], stored biological samples [43], and various biological fluids [44]. It makes miRNAs a promising biomarker for human cancer.

## 3. MicroRNA Expression in Adrenocortical Tumors

The initial evidence of miRNA contribution in adrenocortical tumor pathogenesis came up with the observation that* H19* gene product, previously recognized as a noncoding RNA, would be a miRNA precursor [45]. The* H19* gene is located in 11p15* locus*, the same region of insulin-like growth factor-2 (*IGF2*) gene. The* H19* expression is downregulated in Beckwith-Wiedemann syndrome and is inversely related to* IGF2* [46]. To date, distinct studies reported miRNA expression profile in adults and one in pediatric adrenocortical tumors.

### 3.1. Adrenocortical Tumors in Adults

Tömböl et al. [47] performed miRNA profiling for 368 miRNAs using a quantitative PCR array technique (TaqMan low density array (TLDA)) in 7 ACCs, 9 cortisol producing adenomas, and 10 normal adrenocortical tissue samples. The array revealed significant differences in expression of 22 miRNAs among the experimental groups. Fourteen miRNAs were selected for further validation and significant differences were confirmed in 6 of them: miR-184, miR-210, miR-214, miR-375, miR-503 and miR-511. Of these, miR-184 and miR-503 were overexpressed in ACCs in comparison with normal and benign counterparts and miR-210 was upregulated in ACCs compared with adenomas. Expression of miR-511 and miR-214 was downregulated in ACCs relative to adenomas and normal samples, while miR-375 was upregulated in normal tissues compared with ACCs and ACA. In order to identify potential biomarkers of malignancy, miRNAs with the most significant delta CT (ΔCT) differences between ACCs and the other tissues were tested by receiver-operating characteristics (ROC) curve analysis. When settling the cut-off value of ΔCTmiR-511-ΔCTmiR-503 to 1.4 (diagnosis of ACC if the difference is ≥1.4), ACC could be identified with 100% sensitivity and 97% specificity.

Soon et al. [48] studied 22 ACAs, 27 ACCs, and 6 normal adrenal cortex tissues using the Locked Nucleic Acid microarray technology. When comparing ACCs and ACAs, 23 microRNAs were differentially expressed, 14 upregulated, and 9 downregulated. Interestingly, one sample histologically classified as ACC and grouped together with adenomas in the miRNA profile had remained disease-free seven years after adrenal surgery. This finding suggests that this tumor might be considered as a false positive of the Weiss system. Additionally, primary adrenal and metastatic cervical lymph node from one patient had very similar miRNA expression pattern, suggesting that microRNAs could remain relatively stable between different tumor clones. Three miRNAs were selected for validation using quantitative PCR: miR-195 and miR-335 were significantly downregulated and miR-483-5p had a trend toward being upregulated in ACCs compared with ACAs. Nonetheless, ACC patients with upregulated miR-483-5p and downregulated miR-195 identified a group with poor cancer specific survival. It is noteworthy that miR-483 gene is located within intron 2 of* IGF2*, a gene frequently upregulated in adrenocortical carcinomas [49].

Patterson et al. [50] performed a microarray in 10 ACCs, 26 ACAs, and 21 normal adrenal cortices. Interestingly, the tumors were classified as carcinoma based on clinical characteristics, when gross local invasion or metastasis was identified at any time during the follow-up and not by the usually employed histologic criteria of Weiss score system. Twenty-three miRNAs were differently expressed between ACA and ACC, 5 upregulated, and 18 downregulated. Thirteen miRNAs were chosen for further validation by quantitative PCR and four of them maintained statistical difference between groups: miR-483-5p was upregulated while miR-195, miR-125b, and miR-100 were downregulated in ACCs compared with adenomas. The expression levels of* IGF2* mRNA and miR-483-5p obtained a good correlation, suggesting they were coexpressed from this* locus*. They also demonstrated that an increased expression of miR-483-5p predicted malignancy with 80% sensitivity and 100% specificity.

Schmitz et al. [51] studied 667 miRNAs from 4 primary ACCs, 3 metastases of ACC, 5 cortisol producing adenomas, 4 aldosterone producing adenomas, and 4 normal adrenal cortices. Samples were obtained from formalin-fixed paraffin-embedded tissue. Direct analysis between carcinomas and adenomas revealed 159 miRNAs downregulated and 35 upregulated while comparison between ACCs and APA revealed 38 miRNAs. Three downregulated genes, miR139-3p, miR-675, and miR-335, were validated with quantitative PCR. The authors proposed that expression levels of these miRNAs could differentiate malignant from benign adrenocortical tumors despite the small sample size of the study.

Özata et al. [52] studied 26 ACAs, 22 ACCs, and 4 normal cortex samples by microarray encompassing 903 human miRNAs genes. Unsupervised cluster utilizing the 213 miRNAs, most differently expressed, could reasonably discriminate carcinomas from adenomas and the normal adrenal cortices. Comparison between carcinomas and adenomas revealed 38 miRNAs overexpressed and 17 underexpressed miRNAs. Seven miRNAs were selected for verification on an expanded cohort based on their difference in expression or potential role in adrenal carcinogenesis. In accordance with the microarray results, quantitative PCR analyses demonstrated a significantly high expression of miR-483-5p, miR-483-3p, miR-10, and miR-21 and lower expression of miR-1974, miR-195, and miR-497 in ACCs when compared with adenomas and normal samples. High levels of miR-503, miR-1202, and miR-1275 were persistently associated with shorter survival in ACCs after the analysis of the microarray results and further validation in an expanded cohort. The authors also evaluated the functional consequences from the dysregulation of four miRNAs in a human ACC cell line. Inhibition of miR-483-5p and miR-483-3p led to a significant reduction in cell proliferation. In the same way, a cell transfected with anti-miR-483-3p but not with anti-miR-483-5p showed a significant increase in apoptosis. In addition, miR-483-3p expression level was inversely correlated with the proapoptotic PUMA protein. As expected, the overexpression of miR-195 or miR-497 resulted in a significant decrease in cell growth and a concomitant induction of cell death. Besides confirming results from previous studies and presenting new potential prognostic biomarkers, Özata et al. [52] also clarified the function of miR-483 and miR-195 expanding possibilities for new therapeutic options for advanced ACC patients.

Chabre et al. [53] performed a microarray profile of miRNA in 6 ACAs and 12 ACCs. Twelve miRNAs were differently expressed between adenomas and carcinomas, five underexpressed, and seven overexpressed. Eight miRNAs were chosen for validation in a cohort of 10 ACAs and 18 ACCs. The expression levels of miR-195 and miR-335 were lower in ACCs compared with ACAs. As in other studies, mir-483-5p was markedly upregulated in ACCs.  Table 1 summarizes the main studies that compared microRNA expression profiles in adrenocortical tumors.

### 3.2. Adrenocortical Tumors in Children

Pediatric adrenocortical carcinoma is recognized for its distinct clinical, pathological, and molecular features [54]. To date, there is only one published study that evaluated miRNA expression profile in childhood adrenocortical tumors. Doghman et al. [55] analyzed 25 pediatric adrenocortical tumors and 5 age-matched normal adrenocortical tissue samples. They found 26 dysregulated miRNAs, most of them downregulated in tumors compared with the normal counterpart. Unsupervised cluster analysis of these tumors and normal samples could not clearly discriminate malignant from benign tumors, but three subclusters based on chance of relapse were identified. Not surprisingly, histological classification based on Weiss criteria was not significantly associated with any specific group of samples. Previous evidences indicate that the Weiss system is not entirely applicable to pediatric tumors and does not accurately predict clinical outcome in this population [56]. Interestingly, miR-483-3p was also upregulated in benign and malignant pediatric tumors, which is consistent with the finding that* IGF2* is overexpressed in adrenocortical tumors independently of their malignancy [49]. Functional analysis of miR-99a and miR-100, which were among the highly downregulated miRNAs in tumors, was also performed. Additionally, these miRNAs share the same seed sequence, implying that they could regulate a common set of target mRNAs. It was demonstrated that in adenoma cells these specific miRNAs regulated* IGF1R* and mTOR signaling cascade at multiple levels.

### 3.3. MicroRNA Analysis in Blood Samples

More recently, three studies evaluated microRNAs in blood samples of adrenocortical tumor patients (Table 2). Patel et al. [57] analyzed five miRNAs in serum samples from 17 ACAs and 22 ACCs collected before adrenal surgery. Two of them, miR-34a and miR-483-5p, were found in greater levels in carcinomas than adenomas. The ROC curve analysis revealed a good accuracy for miR-34a (area under the curve = 0.83; *P* = 0.001). There was no association of miRNAs serum expression levels with the extent of disease, positron emission tomography avidity, or disease-free survival time. Szabo et al. [58] demonstrated higher levels of several miRNAs, miR-100, miR-181b, miR-184, miR-210, and miR-483-5p, in plasma samples of a small group of patients with carcinomas when comparing to adenomas. Chabre et al. [53] assessed serum samples of 14 ACAs and 23 ACCs patients using five selected miRNAs. Blood samples from patients with ACC had lower levels of miR-195, miR-335, and miR-376a. On the other hand, miR-139-5p and miR-483-5p exhibited a higher level in the samples of patients diagnosed with adrenocortical carcinoma. The miR-195 was the best biomarker to discriminate malignant from benign tumors (area under the curve = 0.948; *P* < 0.0001), but miR-483-5p was able to differentiate recurring from nonrecurring tumors (area under the curve = 0.929; *P* < 0.0001). Additionally, higher levels of miR-483-5p and lower levels of miR-195 were associated with worse recurrence-free and overall survival.

## 4. Conclusions

The discovery of miRNAs as important regulators of gene expression has expanded our possibilities to understand tumor biology. However, many divergent data from published miRNA profiles in adrenocortical tumors still need to be understood. The correlation between miRNA profiles and the type of the genetic pathways differently activated in adrenocortical tumors, like mutant p53 or abnormal expression of *β*-catenin, would possibly explain some of these divergent findings as it could only reflect the selection of mutually exclusive drivers of tumorigenesis [59]. Among the assured results, miR-483 has the great potential to differentiate adenomas from carcinomas and aggressive from indolent carcinomas, and it might function as a therapeutic target.

## Figures and Tables

**Table 1:** Summary of the current studies that evaluated microRNAs expression profiles in adrenocortical tumors.

Study	Tumors	Dysregulated microRNAs	Major findings
Tömböl et al., 2009 [47]	8 ACAs/4 ACCs/4 normal cortices	**miR-184, miR-210, miR-503** miR-214, miR-511	Difference between dCTmiR-511 and dCTmiR-511 was able to differentiate adenomas from carcinomas

Soon et al., 2009 [48]	27 ACAs/22 ACCs/6 normal cortices	miR-195, miR-335, miR-7	High levels of miR-483-5p and low levels of miR-195 predicted worst overall survival

Patterson et al., 2011 [50]	26 ACAs/10 ACCs/21 normal cortices	**miR-483-5p** miR-195, miR-125b, miR-100	Overexpression of miR-483-5p was able to diagnose carcinomas. The miR-483-5p and *IGF2* were coexpressed

Özata et al., 2011 [52]	26 ACAs/22 ACCs/10 normal cortices	**miR-483-3p, miR-483-5p, miR-210, miR-21** miR-1974, miR-195, miR-497	Downregulation of miR-483-3p and overexpression of miR-195 or miR-497 led to induction cell death *in vitro *

Chabre et al., 2013 [53]	16 ACAs/30 ACCs	**miR-483-5p** miR-195, miR-335	High circulation levels of miR-483-5p or low levels of miR-195 were associated with worst recurrence-free and overall survival

ACAs: adrenocortical adenomas; ACCs: adrenocortical carcinomas; miRNAs in bold are overexpressed and regular ones are underexpressed in carcinomas comparing to adenomas.

**Table 2:** Summary of the current studies that evaluated microRNAs expression profile in blood samples of adrenocortical tumor patients.

Study	Tumors	Dysregulated microRNAs
Patel et al., 2013 [57]	17 ACAs, 22 ACCs	**miR-34a, miR-483-5p**

Chabre et al., 2013 [53]	8 ACAs, 9 ACCs	**miR-139-5p, miR-483-5p** miR-195, miR-335, miR-376a

Szabo et al., 2014 [58]	14 ACAs, 23 ACCs	**miR-100, miR-181b, miR-184, miR-483-5p**

ACAs: adrenocortical adenomas; ACCs: adrenocortical carcinomas; miRNAs in bold are overexpressed and regular ones are underexpressed in carcinomas comparing to adenoma.
